# The Controlled Semi-Solid Fermentation of Seaweeds as a Strategy for Their Stabilization and New Food Applications

**DOI:** 10.3390/foods11182811

**Published:** 2022-09-12

**Authors:** Gabriele Maiorano, Francesca Anna Ramires, Miriana Durante, Ilaria Elena Palamà, Federica Blando, Gianluca De Rinaldis, Ezio Perbellini, Valeria Patruno, Carlo Gadaleta Caldarola, Santa Vitucci, Giovanni Mita, Gianluca Bleve

**Affiliations:** 1 Istituto di Nanotecnologie, Consiglio Nazionale delle Ricerche, 73100 Lecce, Italy; 2 Unità Operativa di Lecce, Istituto di Scienze delle Produzioni Alimentari, Consiglio Nazionale delle Ricerche, 73100 Lecce, Italy; 3 Consorzio Agrario Provinciale Lecce, 73100 Lecce, Italy; 4 Agenzia Regionale per la Tecnologia e l’Innovazione (ARTI)—Regione Puglia, 70124 Bari, Italy; 5 Struttura Speciale Cooperazione Territoriale, Regione Puglia, 70100 Bari, Italy

**Keywords:** edible seaweeds, *Gracilaria gracilis*, microbial starters, safety assessment, nutritional traits, novel food, bioactive compounds, fatty acids, cytoxicity tests

## Abstract

For centuries, macroalgae, or seaweeds, have been a significant part of East Asian diets. In Europe, seaweeds are not considered traditional foods, even though they are increasingly popular in Western diets in human food applications. In this study, a biological processing method based on semi-solid fermentation was optimized for the treatment of the seaweed *Gracilaria gracilis*. For the first time, selected lactic acid bacteria and non-conventional coagulase-negative staphylococci were used as starter preparations for driving a bio-processing and bio-stabilization of raw macroalga material to obtain new seaweed-based food prototypes for human consumption. Definite food safety and process hygiene criteria were identified and successfully applied. The obtained fermented products did not show any presence of pathogenic or spoilage microorganisms, thereby indicating safety and good shelf life. *Lactobacillus acidophilus*-treated seaweeds revealed higher α-amylase, protease, lipase, endo-cellulase, and endo-xylanase activity than in the untreated sample. This fermented sample showed a balanced n-6/n-3 fatty acid ratio. SBM-11 (*Lactobacillus sakei*, *Staphylococcus carnosus* and *Staphylococcus xylosus*) and PROMIX 1 (*Staphylococcus xylosus*) treated samples showed fatty acid compositions that were considered of good nutritional quality and contained relevant amounts of isoprenoids (vitamin E and A). All the starters improved the nutritional value of the seaweeds by significantly reducing the insoluble indigestible fractions. Preliminary data were obtained on the cytocompatibility of *G. gracilis* fermented products by in vitro tests. This approach served as a valid strategy for the easy bio-stabilization of this valuable but perishable food resource and could boost its employment for newly designed seaweed-based food products.

## 1. Introduction

Macroalgae, or seaweeds, are marine bio-resources that many countries are exploring as alternative food sources to accommodate the rapid increases in the global population and the corresponding greater food demand. They offer also a fundamental support for facing the fragility of conventional farming systems and developing risk-resilient diets [1]. Their growth rate is much higher than that of land plants, and their impact on the environment is very limited, since they use renewable living resources, do not require freshwater or fertilizer to grow and are infected by few pests and diseases [2]. Although seaweeds can be collected naturally, cultivation systems are rapidly developing leading to an increase in alga biomass production worldwide that reached 33 million tons (wet weight) in 2016, from which only 0.57% of the volume (0.2 million tons) was produced in Europe [3]. FAO statistics [4] reported that in the decade 2008–2017 the main global suppliers of seaweeds were China and Indonesia (contributing to 91% of the non-EU production), followed by South Korea. This because for centuries, seaweeds have long been a significant part of the East Asian diets and now have become a fundamental source of phycocolloids, a family of heterogeneous polysaccharides with several applications in food, cosmetic, and pharmaceutic industries [5].

In Europe, alga biomass is mainly supplied by Norway (71% of the European production) and then by Ireland and France, mainly by collecting from wild stocks [3]. In contrast, *Porphyra* farming in Japan and China represents a prime example of the large-scale application of agronomical methods to mariculture; together with *Laminaria* cultivation in China and cultivation of Eucheumoid seaweeds around the world, and they represent a gold standard approach of rational and sustainable exploitation of these marine resources [6].

The main species used as food in the Far East are kombu (*Laminaria japonica*), nori (*Porphyra tenera*, *Porphyra yezoensis*), and wakame (*Undaria pinnatifida*) [7]. After harvesting, nori is sun dried or hot air dried and then processed. For kombu preparation, there are very different methods (salting, boiling in soy sauce, rolling, or slicing). Wakame species are traditionally processed by first mixing the algae with ashes and then by drying for 2–3 days on the beach. The obtained processed products are packaged in plastic bags and kept in the dark, and before consumption, they are washed with seawater and freshwater to remove ash and salts. Other species such as green algae, belonging to the genera *Ulva*, *Enteromorpha*, and *Monostroma*, are processed and traded under the name of Aonori.

In the United States, seaweeds are authorized for human food by the Food and Drug Administration, and they are used under the name “kelp” (*Laminaria* sp., *Macrocystis* sp.).

In the European regulations [8], seaweeds are included in the “novel foods” as they are not considered a traditional food, and they represent a marginal market compared with other traditional European foods, although they are increasingly exploited for the production of ingredients and thus becoming popular in western diets [9]. In recent years, some products based on algae have begun to be traded mainly in organic shops.

Seaweeds are a marine resource that do not need land, water for irrigation, or fertilizers for cultivation; they represent a supply of essential nutrients for the production of new functional low-calorie foods [10]. Additionally, seaweeds are rich in several bioactive compounds, such as polyphenols, sterols, alkaloids, flavonoids, tannins, isoprenoids, proteins with essential amino-acids, and polyunsaturated fatty acids, that possess powerful antioxidant and anti-inflammatory properties [9,10,11,12].

In this frame, the fermentation of seaweeds could represent an efficient method of preserving the integrity and safety of a very unstable and perishable harvested wet biomass [10]. This could open the opportunity to develop new food-derived products with improved safety and sensory properties and shelf life and also to obtain new sources of ingredients and new formulations of non-dairy probiotic foods [13]. Additionally, seaweed fermentation can address new challenges such as the provision of alternatives among marketed products, the production of novel foods that can be beneficial for human health, and/or the development of other applications in the animal feed formulation, food safety, and food-coating industries.

Only a few examples of seaweed-based fermented food products have been reported in the literature. Halophilic lactic acid bacteria (LAB) fermentation was successfully applied to produce a high-salt sauce from the protein-rich red seaweed *Pyropia yezoensis* (nori) [14]. The two common North Atlantic brown kelps *Saccharina latissima* and *Laminaria digitata* were fermentable substrates for LAB *Lactobacillus plantarum* [15] and via *Lactobacillus rhamnosus* revealed a potential for developing seaweed-based functional foods [13].

Fermentation experiments were performed on the green alga *Ulva* sp. demonstrating good performance of LAB isolated from fronds left for 17 months after cellulase treatment, in comparison with yeasts and *Bacillus* strains inoculations [16].

A prebiotic effect was reported for extracts of the red alga *Kappaphycus alvarezzi* on *Bifidobacterium* populations [17]. The fermentation with different fungi belonging to the genera *Rhizopus*, *Aspergillus*, and *Trichoderma* was an efficient method of increasing the protein availability of the red seaweed *Palmaria palmata* [18].

Preparations of *Gracilaria* sp., *Sargassum siliquosum*, and *Ulva lactuca* acid and cellulase hydrolysate were tested as substrates for lactic acid production by *Lactobacillus acidophilus* and *Lactobacillus plantarum* since they are able to ferment galactose, the main sugar present in red seaweeds [19] or with a mixed starter containing LAB and yeasts (*L. casei*, *D. hansenii*, and *Candida* sp.) [16].

This research aimed at studying a new stabilization and processing method for seaweed as a possible novel food in the Mediterranean Basin.

As already reported in several studies, different bacterial loads can occur in brown, green, and red seaweeds. Among them, human pathogens can be associated with seaweed in the same densities and compositions as in the surrounding water masses and can negatively affect the shelf life and sensorial quality of the product [20]. At the present time, the market is totally dominated by the traditionally dried seaweed products [4].

Microbial fermentation that uses the growth and metabolic activity of selected microorganisms was here proposed as a novel stabilization alternative to the traditional drying approach. Fermentation, predominantly developed for the stabilization of perishable agricultural products [21], may be also a more suitable processing method for seaweeds species sensitive to physical (thermal and freezing) treatments.

In this study, main safety and quality parameters were chosen among the ones already present in the in-force food EU regulations. For the first time, LAB and non-conventional coagulase-negative staphylococci were used as starter preparations for developing a semi-solid fermentation process [22,23] of the seaweed species *G. gracilis* in which the free liquid content is totally entrapped in the seaweed gel material.

Solid state fermentation cannot be applicable to bacteria since it is generally carried out by fungi, whereas bacteria need for their growth and metabolic activities the high-water activity (a_w_) offered by free-flowing water. Submerged fermentation is generally chosen for bacterial inocula driven processes, even though the ingredients are commonly administered in low and diluted concentrations.

Semi-solid fermentation has been proposed in this study as a compromise to combine the essential free liquid content necessary to ensure nutrient fluxes for bacterial starter strains and an environment in which the seaweed raw material can be used at high concentration level.

The fermentation process of *G. gracilis*, used as a model seaweed species in this study, through the metabolic activities of starter-inoculated microorganisms can both i) stabilize the very perishable seaweed raw material by increasing its food safety and shelf life traits and ii) produce highly nutritious and healthier final products that can represent building blocks for the development of newer, functional food products [10,24,25].

The obtained new products were characterized at the microbiological, chemical, and nutritional levels, and further analyses were carried out in order to assess antioxidant properties, bioactive compound compositions, and fatty acid profiles. Finally, the toxicity and anti-inflammatory traits associated to the treated seaweeds were also preliminary determined by in vitro tests.

## 2. Materials and Methods

### 2.1. Sample Collection and Pre-Treatment

*Gracilaria gracilis* was collected from the Western area of the Lesina lagoon, where a stable coverage of this seaweed was found (41.866470° N, 15.363350° E). About 5 Kg of wet biomass were sampled during April and July 2020. Freshly caught algal biomass was soon transferred to the laboratory, extensively washed with sterile seawater, and immediately used or stored at −40 °C until analyses. For stabilization, the seaweed samples were treated for 3–5 min at 100 °C in drinking water and then cooled at room temperature and finally used for further treatments or frozen at −40 °C.

Bacteria strains used in this study were: a mix of *Lactobacillus sakei*, *Staphylococcus carnosus* and *Staphylococcus xylosus* (SBM-11, Sacco srl, Cadorago, Italy), *Staphylococcus xylosus* (PROMIX 1, Sacco srl, Cadorago, Italy), *Lactobacillus acidophilus* (LA, Prodeco Pharma srl, Castelfranco Veneto, Italy).

### 2.2. Microbiological Analyses

Microbiological analyses of the seaweed were performed as described by Bleve et al. [26] with some modifications. Samples were serially diluted with 1 g/L (*w/v*) peptone water and then applied on agar slants containing these media: for total bacterial count, Plate Count Agar (PCA, Heywood, Lancashire, UK) added with 0.05 g/L nystatin (Sigma-Aldrich, Darmstadt, Germany) and incubated at 30 °C for 48–72 h; for Enterobacteriaceae identification, Violet Red Bile Glucose Agar (VRBGA, LABM, Heywood, Lancashire, UK) incubated at 37 °C for 18–24 h; for coli–aerogenes bacteria detection and enumeration, Violet Red Bile Agar (VRBA, LABM, Heywood, Lancashire, UK) incubated at 37 °C for 24–48 h; for the enumeration of coagulase positive staphylococci, Baird Parker Agar Base (BP, LABM, Heywood, Lancashire, UK) incubated at 37 °C for 24–48 h; for the isolation of pathogenic staphylococci, Mannitol Salt Agar (MSA, LABM, Heywood, Lancashire, UK) incubated at 37 °C for 18–72 h; for the detection and enumeration of *Vibrio* spp., Thiosulfate Citrate Bile Sucrose Agar (TCBSA, Sigma-Aldrich, Darmstadt, Germany) incubated at 37 °C for 18–24 h; for the enumeration of *Bacillus* spp., Bacillus ChromoSelect Agar (BCSA, Sigma-Aldrich, Darmstadt, Germany) added with Polymyxin B supplement incubated at 30 °C for 24–48 h; for hydrogen sulphide producing bacteria, Iron Agar (Lyngby) without cysteine (Sigma-Aldrich, Darmstadt, Germany) incubated at 25 °C for 48 h. The enumeration of yeast and moulds were performed on Dichloran Rose-Bengal Chloramphenicol Agar (DRBC, Thermo Fisher Scientific, Monza, Italy) and incubation at 25 °C for 5 days.

Halophilic microorganisms were identified following the procedure described by Bleve et al. [26] that consisted in plating all samples in: Marine agar (peptone 5 g/L, yeast extract 1 g/L, agar 16 g/L) and R2A (Sigma-Aldrich, Darmstadt, Germany) with the addition of 0.1 g/L of ampicillin and 0.05 g/L of kanamycin and incubation at 25 °C for 2–7 days for fungi and with the addition of 0.05 g/L of nystatin and incubated at 30 °C for 48–72 h for bacteria or; Corn Meal Agar (CMA, Sigma-Aldrich, Darmstadt, Germany) and Sabouraud Dextrose Agar (SDA, LABM, Heywood, Lancashire, UK) with the addition of 0.1 g/L of ampicillin (Merck KGaA, Darmstadt, Germany) and 0.05 g/L of kanamycin (Merck KGaA, Darmstadt, Germany) and incubated at 25 °C for 2–7 days. CMA and SDA media were prepared by the addition of artificial seawater (3% NaCl, 0.07% KCl, 1.08% MgCl_2_, 0.54% MgSO_4_, 0.1% CaCl_2_, *w/v*). For each plate, the number of colonies forming units (CFU) per gram of seaweed was determined.

### 2.3. Molecular Identification of Bacterial Isolates

The bacterial total genomic DNA was extracted by using the Power Soil DNA Isolation Kit (MO BIO; USA) following the manufacturer’s protocol. The 16S rDNA region was amplified according to Bleve et al. [27]. The amplicons were purified and the DNA sequencing was performed as previously described by Bleve et al. [28]. The sequences analysis was made using the Chromas program version 1.45 (www.technelysium.com.au (accessed on1 February 2022)) and the sequence alignment and comparison with the sequences in the GenBank database (Release 233) by BLAST program (https://blast.ncbi.nlm.nih.gov/Blast.cgi?PROGRAM=blastn&PAGE_TYPE=BlastSearch&LINK_LOC=blasthome (accessed on 1 April 2022)).

### 2.4. Seaweed Treatment

Heat-treated seaweeds were ground and homogenized in a blender for 10–15 min by slowly adding water in a final quantity of 150 mL for 100 g of *G. gracilis*. The homogenized product was aliquoted in sterile glass jars of 150 g. The jars were pre-incubated at 35 °C for 15 min and then inoculated with starter microorganisms.

Inocula were prepared in a 1 L flask with 500 mL of MRS medium or nutrient broth for bacterial strains or of Sabouraud broth for yeast strains added with 2% (*w/v*) sea salts (Vibrant Sea™, Seachem Laboratories, Madison, GA, USA). Bacteria strains were inoculated at a final concentration of about 10^7^ CFU/g into sterilized treated seaweed. Three uninoculated samples were also produced as control.

The starter inocula were added to the seaweed puree, accurately mixed, covered with a sterile gauze to ensure the thermal/gas exchanges and finally inserted in large glass jars in order to reduce excessive evaporation. The samples were incubated at 30 °C (*Lactobacillus acidophilus*, Prodeco Pharma srl, Castelfranco Veneto (TV), Italy) and at 37 °C (a mix of *Lactobacillus sakei*, *Staphylococcus carnosus* and *Staphylococcus xylosus* (SBM-11), *Staphylococcus xylosus* (PROMIX 1), *Lactobacillus acidophilus* (LA).

Fermented seaweed samples were aliquoted in 50 g transparent glass jars, submitted to a thermal treatment of 5 min at 90 °C and then stored in the marketplace simulating storage conditions (temperature 25 °C and light exposure, 12 h/day, to an intensity of 500 lux have been electronically controlled), during 30 days with samples safety shelf-life analyses repeated after 7, 15 and 30 days.

### 2.5. Enzyme Activities

Samples were filtered by polyamide filter 355/51 (Saati, Milan, Italy) in order to separate solid and liquid portions

*Enzymatic Activity Assays on Fermented Products.* Activity assays for α-amylase, protease, esterase, lipase, cellulase and endo-xylanase were performed in order to evaluate the production of these enzymes by the microbial starters at the end of fermentation. All experiments were conducted in triplicate.

*Preparation of Crude Enzyme Solution.* Crude enzyme solutions from treated and untreated samples of *G. gracilis* were prepared according to the method of Lee et al. [29] with slight modifications. Briefly, 2.5 g of sample were suspended in 5 mL of distilled water and incubated for 1 h at 30 °C under shaking (1000 rpm). Then, mixtures were filtered by polyamide filter 355/51 (Saati, Milan, Italy), and the resulting liquid portions were centrifuged at 8000× *g* at 4 °C for 15 min. The obtained supernatants were recovered as crude enzyme solutions

*α-Amylase activity assay.* The α-amylase assay was performed with a starch solution as substrate; 72 µL of raw enzyme solution was added to the reaction mixture, which consisted of 50 µL substrate solution (1% potato starch in pH 7 1 M phosphate buffer) and 93 µL of 1 M phosphate buffer at pH 7, the reaction was carried out at 40 °C for exactly 10 min. Then, 714 µL of 0.1 M HCl were added to stop the reaction. Subsequently 15 µL of blocked reaction solution was added to 185 µL of sterile double-distilled water; then, 50 µL of 0.005% iodine solution was added and then measured at 660 nm using nanodrop (Thermofisher). Enzyme control and substrate control were also performed. A standard starch curve was prepared using a 5 mM stock solution, resulting in the following equation: y = 0.7596x − 0.0192. One unit (U) of activity of α-amylase is defined as the amount of enzyme required to release one micromole of glucose reducing-sugar equivalents per minute.

*Protease activity assay*. Protease activity was assayed following the method of Walter [30] and Moyano et al. [31] with the modified procedure proposed by Sigma-Aldrich that employed casein as a substrate. Protease activity was determined by measuring the hydrolysis of 0.66% (*w/v*) of casein in 50 mM Tris-HCl buffer at pH 8. Crude enzyme solutions (50 μL) were mixed with 150 μL of casein substrate at 37 °C. After 30 min of incubation, the reaction was stopped by adding 200 μL of 10% (*w/v*) trichloroacetic acid (TCA), then the mixtures were centrifuged at 8000× *g* for 5 min. An aliquot of 10 μL from supernatants were mixed with 50 μL of 2 M sodium carbonate, 20 μL of Folin and Ciocalteu’s phenol reagent, and 180 μL of water and then incubated at 37 °C for 3 min. Protease activities were measured by recording absorbance at 765 nm. A standard curve was prepared using L tyrosine from 10 mM stock solution, and one unit of enzyme activity was expressed as 1 μmol of tyrosine min^−1^ mg protein^−1^.

*Esterase activity assay.* The carboxyl ester hydrolase (esterase) activities were determined using a spectrophotometric method [32] that monitored the hydrolysis of p-nitrophenylbutyrate (p-NPB) to p-nitrophenol at 37 °C, for 5 min. A stock solution of p-NPB in acetonitrile was added to a final concentration of 10 mM in appropriately diluted crude enzyme solutions (dilution buffer Tris-HCl 0.1 M, pH 7.5). The spectrophotometer was set to zero with Tris-HCl buffer (0.1 M, pH 7.5) and the amount of p-nitrophenol liberated was measured by recording absorbance at 410 nm. A p-nitrophenol standard curve was arranged, and one unit (U) of esterase activity was defined as the amount of esterase needed to liberate 1 μmol of p-nitrophenol per minute from p-NPB under the specified conditions.

*Lipase activity assay.* A spectrometric method that employs p-nitrophenyl palmitate (p-NPP) as a substrate was carried out for the measurement of lipase activities [33]. The substrate solution consists of p-NPP 0.4 mM, sodium dodecyl sulfate 0.6 mM, and Triton X-100 15.5 mM dissolved in distilled water. The assay was prepared by mixing 2.5 mL of substrate solution, 2.5 mL of 0.1 M Tris-HCl buffer (pH 7.5), and 1 mL of properly diluted crude enzyme solution. The release of p-nitrophenol from p-NPP at 37 °C was continuously monitored for 5 min by measuring absorbance values at 400 nm, by using a spectrophotometer. Recorded absorbances were converted to concentration of substrate hydrolyzed by employing a p-nitrophenol standard curve. One unit (U) of lipase activity was defined as the amount of lipase required to release 1 μmol of p-nitrophenol from p-NPP in 1 min, under the corresponding conditions.

*Endo-xylanase activity assay*. The endo-xylanase activity of the supernatants was assayed using Xylanase Assay kits (XylX6 method) (Megazyme, Bray, Ireland) as described by the manufacturer. This assay specifically detects the activity of endoxylanase and not the activity of the enzyme xylosidase or exo-xylanase. The XylX6 reagent contains: XylX6 colorimetric substrate and β-xylosidase. The blocking ketone group at the non-reducing end prevents any hydrolytic action by β-xylosidase on the XylX6 substrate. Incubation with an endoxylanase at 40 °C for 5 min generates an unblocked colorimetric oligosaccharide which is rapidly hydrolyzed by the auxiliary β-xylosidase to quantitatively release the 4-nitrophenol (pNP) which is detected at the absorbance of 400 nm. The endoxylanase activity (one unit) was defined as 1 μmol of pNP released from XylX6 per minute.

*Cellulase activity assay*. Activities of microbial cellulases were determined using a cellulase assay kit (CellG5, Megazyme, Bray, Ireland), according to the method provided by the manufacturer. Briefly, properly diluted crude enzyme solutions were incubated with CellG5 solution that contains β-glucosidase and 4-nitrophenyl-β-D-cellopentaoside (BPNPG5) at 40 °C, for 5 min. BPNPG5 was specifically hydrolyzed by cellulase, and the hydrolysate was subsequently degraded by β-glucosidase present in the substrate mixture, thus releasing 4-nitrophenol (pNP) that was spectrophotometrically detected by measuring the absorbance at 400 nm. The cellulase activity (one unit) was defined as 1 μmol of pNP released from CellG5 per minute.

### 2.6. Physicochemical Analyses

Salinity, pH and water activity were evaluated at day 0 and after three days treatment. Salinity was measured by using a salinity refractometer for seawater and marine aquaria 0–10% hydrometer with automatic temperature RHS-MR110 ATC (Agritechstore, Mori, Trento, Italy). For the biochemical analyses reported below seaweed samples were freeze-dried using an Alpha 2–4 LSC plus freeze-dryer (Martin Christ Gefriertrocknungsanlagen GmbH, Osterode am Harz, Germany) with a vacuum pressure of 0.015 mbar and a condenser temperature of −60 °C. The freeze-dried samples were ground at 500 µm by using a Retsch laboratory mill (Torre Boldone, BG, Italy) to obtain a homogeneous powder.

### 2.7. Total Polyphenol Content Analyses and Antioxidant Capacity Methods

Seaweed samples were extracted in triplicate following the methodology reported by Capillo et al. [34], with some modifications. Briefly, 200 mg dried seaweeds were macerated with 10 mL 80% methanol (*v/v*) at 4 °C, over-night. After centrifugation for 10 min at 2000× *g*, the supernatant was collected, further 10 mL of extraction solvent was added to the pellet, and the extraction was repeated on a rotary shaker for one hour. Pooled supernatants were concentrated by evaporation at 35 °C in a rotavapor (model R-205 Büchi, Büchi Labortechnik AG, Switzerland) and re-suspended in MilliQ-water to at a concentration of 50 mg/mL. Extracts were filtered on 0.45 µm CA syringe filter (Filtres Fioroni, France), portioned and stored at −20 °C before the HPLC analysis and antioxidant activity assays. The extraction experiments were performed twice, with each triplicated extraction considered for HPLC injection. The HPLC-DAD separation and identification of polyphenols were performed using the same chromatographic method and column as already reported [35]. The extracts were assessed for total phenol content (TPC) and antioxidant capacity using the ABTS assay (Trolox Equivalent Antioxidant Capacity–TEAC) as already reported [35]. A rapid microplate methodology, using a microplate reader (Infinite M-200, Tecan Group Ltd., Männedorf, Switzerland) and 96-well plates (Costar, 96-well clear round bottom plate, Corning) was used.

### 2.8. Extraction and Quantification of Proteins

Protein from seaweeds were extracted and quantified as reported by Barbarino and Lourenço [36], with slight modifications. Fifty mg of dried seaweed were dispersed in 4 mL of ultrapure water, grinded by using POLYTRON® PT 10–35 GT (Kinematica AG, Switzerland) and incubated at 4 °C for 6 h on a rotating wheel. Then, dispersions were centrifuged at 12,000 g for 20 min at 4 °C, supernatants were collected and extraction was repeated as above described. Residues were added of 1 mL 0.1 M NaOH supplemented with 0.5% β-mercaptoethanol (*v/v*), these mixtures were incubated at 4 °C for 1 h on rotating wheel and then, samples were centrifuged at 12,000 g for 20 min at 4 °C. The collected supernatants were combined to the previous extractions to obtain a final volume of 9 mL. In order to precipitate proteins, each extract was added of 27 mL of 25% (*w/v*) trichloroacetic acid (TCA) at 4 °C. The mixtures were kept at 4 °C for 1 h and then centrifuged at 16,000 g for 20 min at 4 °C. After discarding supernatants, precipitates were washed with 10% of cold TCA and centrifuged again. Pellets were recovered, washed with ice-cold acetone and let to air-dry, resuspended in 0.5 mL of NaOH 1 M and the protein contents were quantified by using Bradford assay (Sigma-Aldrich), following the manufacturer’s instructions. A calibration curve was prepared using bovine serum albumin (BSA) (Sigma-Aldrich) in a range of 0–865 µg/mL. Samples were arranged in a 96-well plate (Corning) together with standards and blanks and nteroba for their absorbance at 595 nm by using a microplate reader (CLARIOstar PLUS, BMG LABTECH). Protein extractions were performed six times for each sample.

### 2.9. Determination of Insoluble Indigestible Fraction (IIF) of Seaweeds

The extraction and quantification of the indigestible fraction of the seaweeds was performed as reported by Rupérez and Toledano [37], with slight modifications. One hundred mg of dry weight (DW) seaweed were dispersed in 10 mL of hydrochloric acid-potassium chloride buffer (0.05 M HCl/0.03 M KCl, pH 1.2) containing 40 mg of pepsin from porcine gastric mucosa (Sigma-Aldrich, ≥250 units/mg), the dispersions were incubated at 40 °C for 1 h with constant mixing. Then, 19 mL of 50 mM sodium phosphate pH 6.9 were added and the pH carefully checked, and 1 mL of 100 mg/mL α-Amylase from porcine pancreas in 20 mM sodium phosphate, 6.7 mM sodium chloride, pH 6.9 was added. Samples were incubated at 37 °C for 15 h under constant mixing. After incubation, samples were centrifuged at 10,000 g for 15 min, residues were washed twice with 5 mL of ultrapure water and then, they were dried at 105 °C for the following gravimetric determinations of the insoluble indigestible fraction. Extractions were performed six times for each sample.

### 2.10. Lipid Extraction and Fatty Acids Analysis

Lipids were extracted from 200 mg of dried samples with 5 mL of n-hexane under mechanical stirring (300 rpm) at 4 °C for 16 h. Samples were centrifuged at 4500 g for 5 min and the organic phase was recovered and evaporated to dryness under a stream of nitrogen.

The quantity of lipid extract was calculated by the following equation:

Lipid content (%) = DW of extracted lipid (g)/DW sample (g) × 100.

Fatty acid derivatization was carried out according to Durante et al. [38] with some modifications. Briefly, total lipids were saponified at 90 °C for 5 min with a methanolic solution (3 mL) of 0.5 M KOH. After cooling, BF_3_ in methanol (2 mL, 12% *w/v*) was added and samples were incubated at 100 °C for 30 min. One mL of n-hexane and 1 mL of sodium chloride (0.6% *w/v*) were added to the reaction mixture, and it was vigorously stirred for 30 s and the organic upper phase was analysed according to Durante et al. (2016) using an Agilent 5977E GC/MS system equipped with an Agilent DB-WAX column (60 m, 0.25 mm i.d., 0.25 mm film thickness).

### 2.11. Calculation of Nutritional Indices for Assessing Fatty Acids

Fatty acid quality was determined using the PUFA to SFA (P/S) ratio, n-6/n-3 fatty acids ratio, the index of atherogenicity (IA), index of thrombogenicity (IT), h-hypocholesterolemic/H-hypercholesterolemic (h/H) ratio and unsaturated index (UI) according to Chen and Liu [39]. The indices were calculated using the equations below:

IA: [C12:0 + (4 × C14:0) + C16:0]/UFA
IT: (C14:0 + C16:0/[(0.5 × MUFA) +(0.5 × n-6 PUFA) + (3 × n-3 PUFA) + (n-3/n-6)]
h/H: (MUFA + PUFA)/(C14:0 + C16:0)
UI: 1 × (% monoenoics) + 2 × (% dienoics) + 3 × (% trienoics) + 4 × (% tetraenoics) + 5 × (% pentaenoics)

where:

PUFA = polyunsaturated fatty acids
SFA = saturated fatty acids
UFA = unsaturated fatty acids
MUFA = monounsaturated fatty acids

### 2.12. Isoprenoids Content and Analysis

Isoprenoids (tocopherols, carotenoids and chlorophylls) were extracted on 50 mg of freeze-dried and analysed by HPLC-DAD as described by Durante et al. [40] using an Agilent 1100 Series HPLC system equipped with a reverse phase C30 column (5 µm, 250 × 4.6 mm) (YMC Inc., Wilmington, NC, USA). The detector was set at 290 nm, 450 nm and 665 nm for total tocopherols, carotenoids, and chlorophylls, respectively.

### 2.13. Cell Culture and Preparation of Aqueous Extract of Seaweeds

Human Burkitt lymphoma *Namalwa* cell line were maintained in RPMI supplemented with 10% FBS, 100 U/mL penicillin, 100 mg/mL streptomycin, 5% L-glutamine and 5% sodium pyruvate in a humidified incubator at 37 °C, 5% CO_2_ and 95% relative humidity. Cells were regularly tested for *Mycoplasma* contamination.

Aqueous extracts of seaweeds were prepared for biological evaluations. One hundred mg of freeze-dried sample were dispersed in 15 mL of ultrapure water (1.5% *w/v*) and incubated at room temperature for 6 h on a rotating wheel. Then, dispersions were centrifuged at 12,000 g for 20 min at 4 °C, supernatants were individually filtered with 0.45 µm PVDF syringe filters and then freeze-dried. Resulting powders were weighted and resuspended in ultrapure water to obtain dispersions concentrated 2 mg/mL. Prior in vitro testing, the aqueous extracts were filtered through 0.22-μm sterile syringe filters.

### 2.14. Cell Viability Assay

*Namalwa* cells (10^5^ cells/well) were seeded in 48 well plate, 200 µg of seaweed extract was added in each well and then cells were incubated in a humidified incubator at 37 °C, 5% CO_2_ and 95% relative humidity for 24 h. Untreated (CTR) samples (10^5^ cells/well) were used as the control groups, 1 h prior the viability assay cells lysed by 1% Triton-X-100 were used as the positive control. MTT assay was employed to evaluate cell viability, briefly, after incubation with extracts, MTT solution was added to cultures, plate was then incubated for 3 h at 37 °C, 5% CO_2_ and 95% relative humidity. Then, the resulting MTT formazan crystals were dissolved with acidified isopropanol. The absorbance was spectrophotometrically measured at wavelength 570 nm by using a microplate reader (CLARIOstar PLUS, BMG LABTECH). The cell viability is expressed as the relative growth rate (%RGR) by following equation:

RGR = D_sample_/D_control_ × 100 (1)

where D_sample_ and D_control_ were the absorbances of the sample and the negative control.

### 2.15. Cell Cycle Investigation

*Namalwa* cells were seeded and treated with seaweed extracts as already described for cell viability assay. After incubation with seaweed extracts (24 h), cells were harvested and washed in PBS by centrifuging at 850 g and gently discarding the supernatant. Then, cells were fixed by adding drop wise to the pellet 200 µL cold 70% ethanol (*v/v*) while vortexing to minimize clumping, fixation was performed for 30 min at 4 °C. After, cells were washed two times in PBS and treated with 5 µg of RNase (stock solution 100 µg/mL). For staining DNA, 100 µL propidium iodide (PI) were added (50 µg/mL stock solution). The cell cycle distribution was determined by analyzing 10,000 ungated cells using a Beckman-Coulter CytoFLEX S.

### 2.16. Cellular ROS Detection Assay

Cytofluorimetry analyses of cellular ROS were performed by analyzing 10,000 ungated cells treated/untreated with seaweed extracts in a Beckman-Coulter CytoFLEX S. Briefly, *Namalwa* cells (10^5^ cells/well) were treated with different seaweed extracts (200 µg/well) for 24 h at 37 °C and 5% CO_2_. After incubation, cells were washed in PBS and stained with ROS detection reagent (Abcam) according to the manufacturer’s instructions. Cellular ROS were determined by analyzing 10,000 ungated cells by using a Beckman-Coulter CytoFLEX S.

### 2.17. Statistical Analysis

All data represent the mean of at least three independent replicates (*n* = 3).

Statistical analysis was assessed by performing both a parametric method (two sample-t-test and one-way ANOVA with post hoc Tukey’s test for multiple groups comparison) and a nonparametric one (Mann–Whitney U Test) by means. All statistical comparisons were performed using Sigma-Stat, version 3.11 (Systat Software Inc., Chicago, IL, USA).

## 3. Results

The cultivation and use of *Gracilaria* spp. have been promoted in several countries, including East and Southeast Asia; Chile and Brasil in South America; and also Morocco, Tunisia and Spain, essentially for agar production demand [41]. The red alga *Gracilaria gracilis* was reported as one of the most naturally abundant species in the Lagoon of Venice and in the Lesina Lagoon (southern Adriatic Sea), and several attempts were launched for its cultivation on field [42].

**Figure 1:**
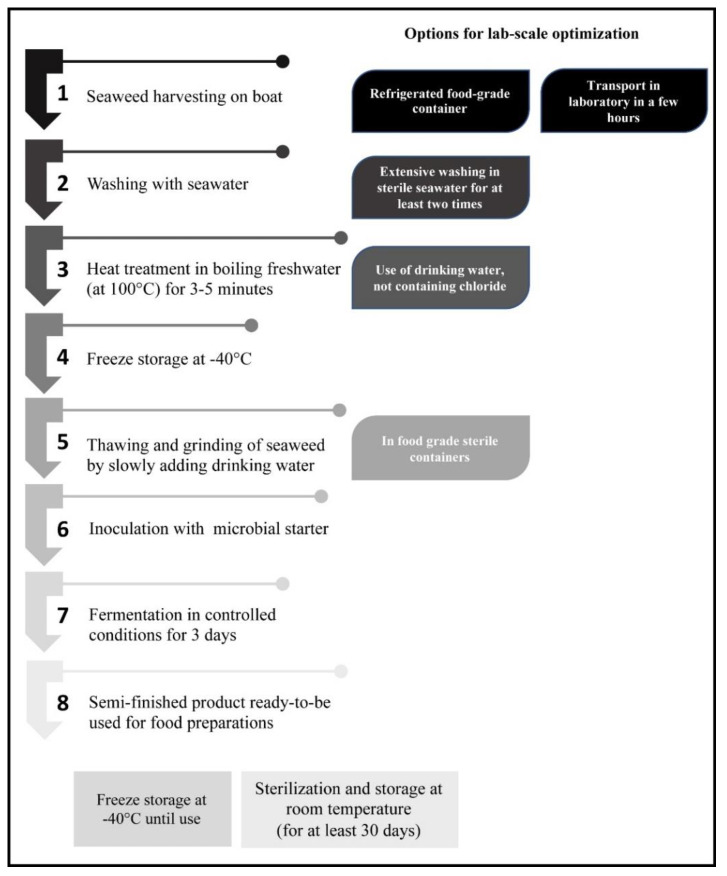
Diagram illustrating the procedure of the new proposed seaweed treatment method.

In this study, a new procedure for the fermentation of the seaweed *G. gracilis* was investigated to obtain food products. The main phases proposed for seaweed preparation are shown in Figure 1, step by step from the starting material to semi-finished food products.

Three different microbial starter fermentation strategies were approached using commercially available preparations: *Lactobacillus acidophilus* (Prodeco Pharma srl, Castelfranco Veneto, Italy); *Staphylococcus xylosus* (PROMIX 1, Sacco srl, Cadorago, Italy); a mix of *Lactobacillus sakei*, *Staphylococcus carnosus* and *Staphylococcus xylosus* (SBM-11, Sacco srl, Cadorago, Italy). Although seaweeds were tested as ingredients added in media for the LAB strains growth [13,15], at the best of our knowledge, no applications concerning the use of non-conventional starter cultures of coagulase-negative staphylococci for fermenting seaweed have been reported.

The use of *Lactobacillus acidophilus* was proposed for seaweed fermentation since strains belonging to this species are usually used to inoculate dairy (milk, yogurt and kefir) and non-dairy (soy milk, fermented vegetables/fruits juices and fermented meat) products. The metabolic activity of *Lb. acidophilus* can be involved in the production of organoleptic properties for fermented foods and inhibits foods spoilage, especially by the production of bacteriocins [43].

Commercial starter preparations of non-conventional staphiloccocci, containing a single strain or a mix of different strains, were proposed here for their robustness to many environmental stresses (such as salt concentration) and their versatile metabolism [44], which makes them very promising for different seaweed raw materials ranging from low protein/high carbohydrates content to high protein/low carbohydrates ones. Non-conventional staphylococci are recently included in commercial starters preparations to produce animal-derived fermented food preparations based on fish, cheese and meat [44]. The protein content of *Gracilaria* ranges from 5 to 45% DW, thus it represents a possible good niche for testing staphylococci starter strains [45]. Moreover, selected strains held mechanisms to manage several environmental stresses (osmotic, oxidative, pH) and revealed versatile metabolic aspects in their metabolism, in this study their use was proposed for facing the seaweed as unique source for their growth and metabolism.

*Microbiological analyses of fermented seaweeds.*

None of the tested samples revealed the presence of bacterial pathogens belonging to *Clostridium perfringens*, pathogenic staphylococci and *Vibrio* spp. (Table 1).

In addition, the search for potential spoilage microorganisms, such as hydrogen sulfide-producing bacteria (such as *Pseudomonas* spp. and possibly *Shewanella putrefacens/Aereomonas hydrophila*), coli-aerogenes bacteria and enterobacteriaceae produced negative results in all tested samples. In addition, with the exception of a very low count in the LA inoculated sample, yeasts and moulds were undetectable in all seaweed obtained products.

In the starter-inoculated samples, the total bacterial count matched with the data obtained on sMRS-glucose for LA (used for lactic acid bacteria) and on sMRS-Sucrose (used for evaluating the presence of staphylococci) for PROMIX 1 and SBM-11. The levels of *Bacillus* spp. were lower than total bacterial count and *Bacillus cereus* was not revealed in any tested sample. Only the uninoculated control contained the highest levels of total bacteria almost belonging to *Enterococcus* spp., *Bacillus* spp. and *Lysinibacillus* spp., as confirmed by the molecular identification of 16S rRNA sequence performed on thirty different colonies isolated from PCA, sNA and BCSA media (data not shown).

Then, the most important quality and safety parameters to be applied to seaweed-based foods were chosen. Many food safety and process hygiene criteria were borrowed from the law in force in terms of multi-ingredient cooked and uncooked ready-to-eat preparations in compliance with European Union laws. All the selected standards parameters considered in the current study are listed in Table 2. These were applied to analysing the effects produced on seaweed *G. gracilis* after the inoculation of different commercial formulation of bacteria.

The effect of long-term storage in environmental conditions of heat-treated fermented seaweed products was evaluated by shelf-life analyses of the profile of pathogenic and spoilage microbiological species for 30 days. All the starter-inoculated samples together with the uninoculated ones did not show presence of pathogenic and spoilage microorganisms until 30 days storage (data not shown). However, the uninoculated control sample prepared without the addition of bacterial starters produced a very unpleasant pungent intense and unacceptable odour and was considered rotten.

The pH started at 8.6 and then quickly decreased (4.9–5.82) within the first 24 h of fermentation at 30 or 37 °C, and after 72 h, pH reached its minimum values, with the exception of the uninoculated sample, which showed a slight increase to 6.02 (Table 3). Two of the three inoculated samples showed pH < 4.2. Salinity values, checked throughout the fermentation, started from 2 to a slight decrease during the 72 h incubation. The water activity tested at 26 °C was about 0.96 for all the tested samples at the end of fermentation.

*Enzyme activities of fermented seaweeds.*

**Figure 2:**
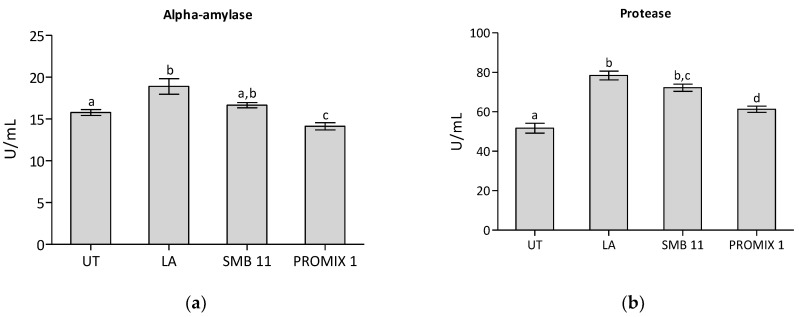

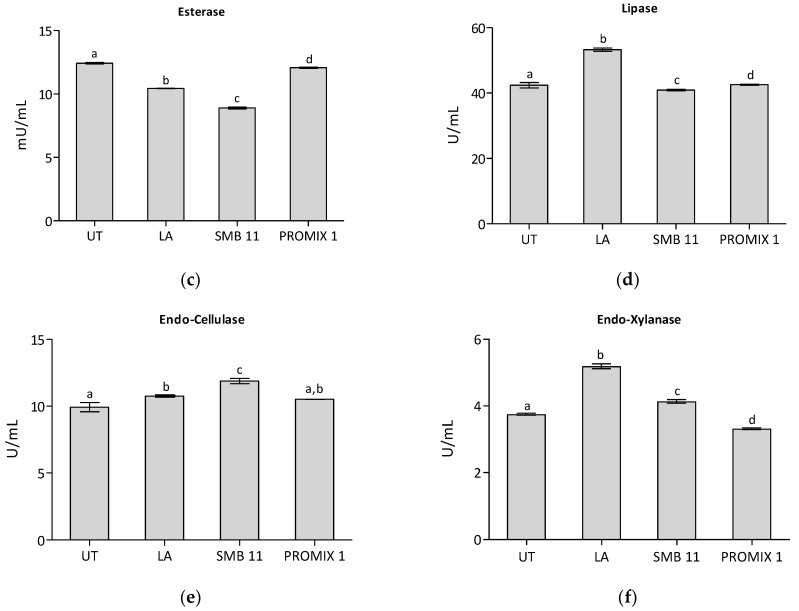
Enzyme activities in untreated control (UT) and in fermented seaweed. LA: *Lactobacillus acidophilus* SBM-11: a mix of *Lactobacillus sakei*, *Staphylococcus carnosus* and *Staphylococcus xylosus*; PROMIX 1: *Staphylococcus xylosus*. Data were submitted to one-way analysis of variance (ANOVA), different letters: Tukey’s post hoc method was applied to establish significant differences among samples (*p* < 0.05).

Enzyme activities measured in various *G. gracilis* samples were reported in Figure 2. The heat-treated seaweed sample showed activities in lipase (42.42 units/mL), esterase (12.42 units/mL), protease (10.34 units/mL), endo-cellulase (9.92 units/mL), endo-xylanase (3.75 units/mL), amylase (15.78 units/mL). All fermented samples revealed higher protease and endo-cellulase activities and a lower esterase activity in comparison with the UT.

Although during food fermentations enzyme activities are predominantly ascribed to endogenous enzymes [64,65], each fermentation driven sample produced a distinct enzymatic profile, with the highest levels of lipase, protease, amylase and endo-xylanase activities in the LA-treated one, and the highest level of endo-cellulase activity in the SBM-11 sample.

The enzyme activities registered are the sum of the two components within each sample, those deriving from the seaweed tissues and those produced by the microbial counterpart. During the fermentation process, the enzyme concentrations and stability can change depending on several abiotic and biotic factors: temperature, pH, microbial consortia evolution, production of potential inhibitors, etc. However, as reported in Figure 2, LA-treated samples revealed α-amylase, protease, lipase, endo-cellulase and endo-xylanase higher than in the untreated sample. In the SBM-11 treated sample, there were higher protease, endo-cellulase and endo-xylanase activities than in the untreated one.

In *L. acidophilus*, the presence of activities attributable to cellulase and hemicellulase enzymes were already reported in the use of banana carbohydrates as carbon source, whereas the growth on sweet potato matrix was supposed to be due to its capacity to produce exogenous enzymes such as amylase, protease, and lipase [66].

As expected, coagulase-negative staphylococci displayed proteolytic and lipolytic activities [67], but the high levels of endo-cellulases and endo-xylanases may probably be ascribable to the presence in the SBM-11 starter mix of the *L. sakei,* which showed a xylooligosaccharides metabolism [68].

Higher enzyme activities in fermented seaweeds can be regarded as a good indicator of active metabolism carried out by microbial starters during fermentation of a source far from their usual living habitat. The bacterial enzymes can be also advantageous for degrading raw material and making it suitable for further food and nutraceutical applications.

As already demonstrated for other fermented food products, different enzymatic activities, such as α-amylase, protease, lipase, and esterase, can be also in turn directly and/or indirectly related to the formation of volatile components during fermentation [69].

*Nutritional analyses.*

**Figure 3:**
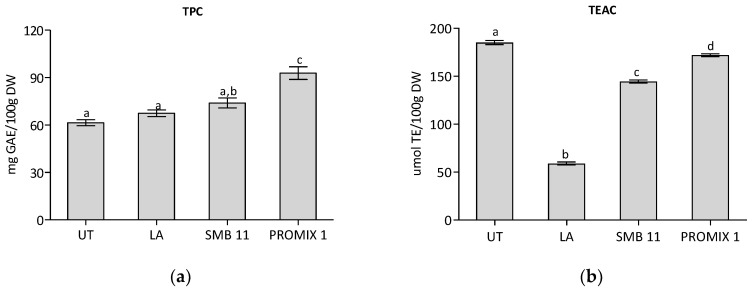
Total Phenol Content (TPC) and Trolox Equivalent Antioxidant Capacity (TEAC) in untreated control (UT) and in fermented seaweed. LA: *Lactobacillus acidophilus* SBM-11: a mix of *Lactobacillus sakei*, *Staphylococcus carnosus* and *Staphylococcus xylosus*; PROMIX 1: *Staphylococcus xylosus*. Data were submitted to one-way analysis of variance (ANOVA), different letters: Tukey’s post hoc method was applied to establish significant differences among samples (*p* < 0.05).

Chromatographic analysis of seaweed methanolic extracts showed few and quite negligible peaks at 280 nm, probably because polyphenols had been dissolved in the boiling water (as a raw material stabilization treatment). Additionally, the Folin–Ciocalteu assay gave a low TPC value, both for untreated control (UT) and treatments. Treatment with PROMIX 1 gave a statistically highest TPC value (Table 4). In this study, seaweeds were heat-treated to reduce the presence of epiphytic microorganisms and other associated organisms. This step probably affected the phytochemical content and antioxidant properties of the raw material, probably due to the leaching of nutrients in the water, during heating process. In fact, Francavilla et al. [45], showed that the water (at 80 °C) produced a richer extract in polar compounds (i.e., polyphenols, carbohydrates and organic acids). However, TPC obtained in all samples were comparable to literature-reported values [13]. Probably the microbiological intervention released new phenol compounds which reacted with Folin–Ciocalteu reagent, as in the case of PROMIX 1. TEAC was greatly or slightly reduced after the treatment with LA, SBM-11 and PROMIX 1, respectively. Even if the phenolic content increases during fermentation (with the PROMIX 1 starter intervention), the antioxidant capacity of untreated samples is similar but statistically higher than in fermented samples (compared to PROMIX 1). The higher TEAC value in the untreated control was probably associated with the presence of non-phenolic compounds with antioxidant activities (for example phycobiliproteins, which are much present in red seaweeds) [70,71] (Figure 3).

During fermentation, microbial metabolic activities can strongly influence the starting phenol compounds profiles, mainly due to several different hydrolysis events, and thus produce a release of simpler phenolics from the plant tissues [72,73]. It can be hypothesized that enzymes of microbial origin contribute to protein complex dissociation to yield proteins in only the monomer form, with different antioxidant activity level.

Generally, the lipid fraction of seaweeds represents 1–6% of dry weight (DW) [74,75,76]. Despite the low lipid content, seaweeds are considered an important source of polyunsaturated fatty acids (PUFA). In particular, some seaweeds contain essential fatty acids such as linoleic and α-linolenic acids. In this work, lipid content in *Gracilaria gracilis* was ~5%. Francavilla et al. [45] and Rosemary et al. [77] in *G. gracilis* and *corticata* reported values of 2% and 7%, respectively. After treatment for 72 h, lipid level in PROMIX 1 was lower than UT. As concerned the fatty acid composition, in UT the most abundant fatty acids identified were arachidonic acid (~46% of TFA) and palmitic acid (~22% of TFA). PUFA were ~55% of TFA, followed by saturated fatty acids (SFA) (~34%) and monounsaturated fatty acids (MUFA) (~10%). Similar results were reported by Afonso et al. [78] who evaluated the fatty acid profile in *Gracilaria* harvested seasonally, observing in the spring–summer an increase in total PUFA and a concomitant decrease in SFA.

After treatment with LA, PROMIX 1 and SBM-11 was observed a statistically significant increase in SFA and a decrease in MUFA and PUFA compared to not fermented (UT) sample was observed.

Dietary intake of fatty acids plays important roles either in the cause or the prevention of cardiovascular disease (CVD) [79]. Generally, dietary SFA, TFA and/or improper ratio of n-3/n-6 fatty acids are common causes of CVD [80,81]. In this work, the nutritional and health indicators such as P/S ratio, n-6/n-3 ratio, IA, IT, h/H and UI were used to assess the nutritional quality of fatty acids.

P/S ratio is an important parameter used to assess the nutritional value of dietary foods such as seaweed, meat, fish, shellfish and food products. For a “balanced diet” the suggested P/S ratio suggested is above 0.4–0.5 [82]. P/S ratio for *G. gracilis* before and after treatment largely exceeded the suggested values, except for LA sample (Table 4). A balanced n-6/n-3 fatty acid ratio has positive effects on decreasing the risk of CVD and cancers. According to the WHO, the n-6/n-3 ratio must be lower than 10 [45]. It must be considered that biochemical composition of seaweeds depends on many environmental and seasonal factors [83,84]. Our results on n-6/n-3 ratio, except for LA sample, are higher than 10 because of the high concentration of arachidononic acid. Similar results were observed by Francavilla et al. [45], who reported that in *G. gracilis*, the amount of fatty acids changes with the season; in particular, the n-6/n-3 ratio was lower in April but increased more than 10 times in January.

IA and IT characterize the atherogenic and thrombogenic potential of fatty acids, respectively. Although the recommended values for the IA and IT are not yet precisely defined, a fatty acid composition with IA and IT less than 1 is considered of good nutritional quality [85]. Our results, except for the LA sample, showed IA and IT both below 1.

Likewise, the h/H ratio is an index of the effects of fatty acids on cholesterol. A greater h/H ratio is directly proportional to a high PUFA content, which is beneficial for human health. *G. gracilis* presented an h/H value of 2.52 similar to the value reported by Afonso et al. [78] in the same species collected on March. Furthermore, after 72 h of fermentation, SBM-11 presented the highest h/H value with difference not significant compared to the UT.

UI is commonly used for judging the content of high-quality PUFA of seaweeds. Usually, the UI values of seaweeds varies widely from 45 to 368.68, and it may be closely related to the species [39]. In this work, after fermentation, the UI ranged from 59.68 in LA to 201.81 in SBM-11, the latter one presents a high degree of total unsaturation such as UT. Similar results were reported by Kumar et al. [86] and Kumari et al. [87], who observed that UI values in Rhodophyta ranged from 50.63 to 250.

Red seaweeds are a valuable source of bioactive compounds with important nutritional functions. However, the nutrient profile of seaweeds is influenced by different factors such as seaweed species, habitat, maturity stage, season and water temperature [88,89]. The isoprenoid content of *Gracilaria gracilis* used as control and fermented seaweeds is shown in Table 5. The α-tocopherol (α-T) level, the most biologically active form of the vitamin E, was reduced from 3.90 mg/100 g DW to 1.56 in SBM-11, 0.38 in PROMIX 1, and it was completely cleared in LA. With respect to the carotenoid content, the level obtained in *Gracilaria gracilis* (64.59 mg/100 g DW) resulted higher than those previously reported for other *Gracilaria* species [77]. Although a high reduction in the total carotenoid content was observed after treatment, the fermented seaweeds can still be considered an important source of such antioxidant molecules, especially for zeaxathin, α-criptoxanthin, β-criptoxanthin and β-carotene. A similar trend was observed for chlorophylls (a+ b) which were higher in UT and in fermented seaweeds than was reported usually for red seaweed [39,77].

As already reported, *Gracilaria* showed a protein content ranging from 5 up to 45%, on dry weight [45]; in this frame, the quantification of the water extractable proteins in treated/untreated seaweeds represented a useful investigation to evaluate the influence of semisolid fermentation on of the release of resistant proteins that are tightly associated to cell walls, being refractory to water extraction [37]. As shown in Figure 3, samples treated with LA reported a content of water-extractable proteins that is statistically similar to UT, on the contrary seaweed fermented by employing SBM-11 and PROMIX 1 starters increased the amount of extractable proteins up to 6.6% and 11.3% on dry weight. These results are related to the different enzyme activities registered in *G. gracilis* treated samples that could act both in releasing resistant protein from seaweed matrix and in modifying the amount and/or the physical–chemical properties of co-extracted of phycocolloids, that are anionic polysaccharides in the cell walls of seaweeds that could trap proteins in their macrostructures during extraction [36].

**Figure 4:**
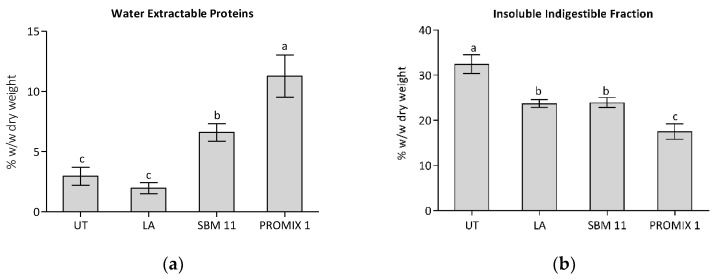
Determinations of water extractable proteins and insoluble indigestible fraction on untreated control (UT) and in fermented seaweed LA: *Lactobacillus acidophilus*; SBM-11: a mix of *Lactobacillus sakei*, *Staphylococcus carnosus* and *Staphylococcus xylosus*; PROMIX 1: *Staphylococcus xylosus*. Data were submitted to one-way analysis of variance (ANOVA), different letters: Tukey’s post hoc method was applied to establish significant differences among samples (*p* < 0.05).

The evaluation of the insoluble indigestible fraction (IIF) reported in Figure 4 shows a statistically reduction of the IIF in samples fermented with microbial starters. The higher level of endo-xylanases and cellulases activities introduced in seaweed samples trough fermentation are responsible for the statistically reduction of the IIF, in particular in PROMIX 1 treated samples. The insoluble indigestible fraction comprises resistant proteins, minerals and insoluble non-starch polysaccharides. The reduction of IIF trough fermentation represents a valuable strategy to improve nutritional value of seaweeds, especially for the decrease of resistant proteins that are inaccessible to proteolysis of seaweed proteins, since protein–polysaccharide interactions within the algae matrix are able to hinder the formation of enzyme–substrate complexes [90].

Seaweeds are characterized for a content of polysaccharides that ranges from 4% to 76% of their total dry weight. The use and demand of these compounds, which are very important in nutraceutical and pharmaceutical industries, is rapidly increasing, with a consequent rise of their commercial value [91]. Moreover, polysaccharides from seaweed are deeply studied for applications in advanced biomedical fields as biocompatible, natural polymer materials [91], and as bioactive agents with several biological properties [92]. Polysaccharides from different species of *Gracilaria* have been tested for their immunomodulatory [93], antiviral [94,95], antiproliferative [96], anti-protozoan [97] antioxidant and anticoagulant [98] activities.

*In vitro testing of fermented seaweeds.*

**Figure 5:**
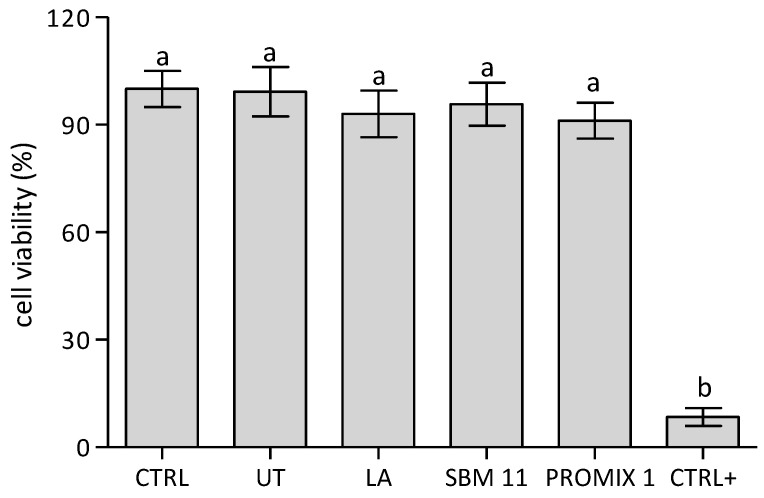
MTT cytotoxicity tests on *Namalwa* cells treated with 200 µg of seaweed water extracts for 24 h. Results are compared to untreated cells (CTR) and cells lysed with 0.1% Triton X-100 (CTR+). Representative measurements of three distinct sets of data. Data were submitted to one-way analysis of variance (ANOVA), different letters: Tukey’s post hoc method was applied to establish significant differences among samples (*p* < 0.05).

**Figure 6:**
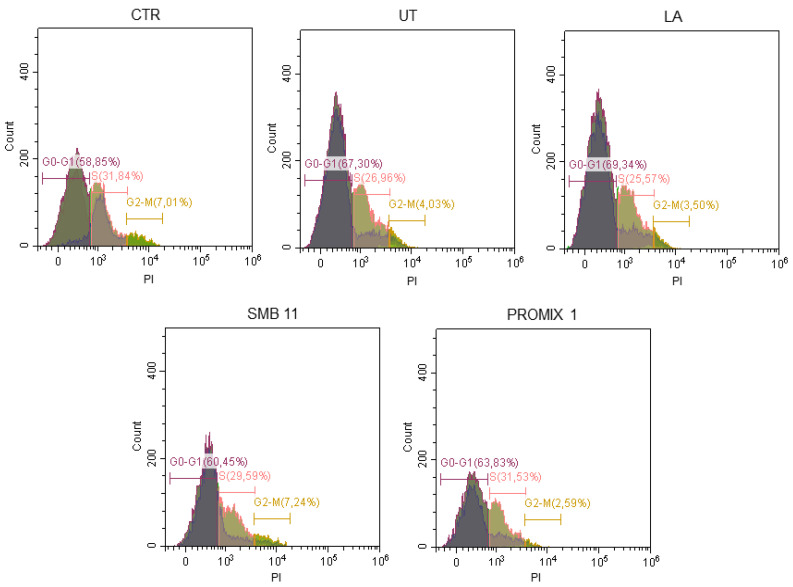
Cytofluorimetric analysis of cell cycle of *Namalwa* cells after incubation with seaweed extract (UT, LA, SBM-11, PROMIX 1) for 24 h compared with untreated control cells (CTR). Representative images of three independent experiments.

**Figure 7:**
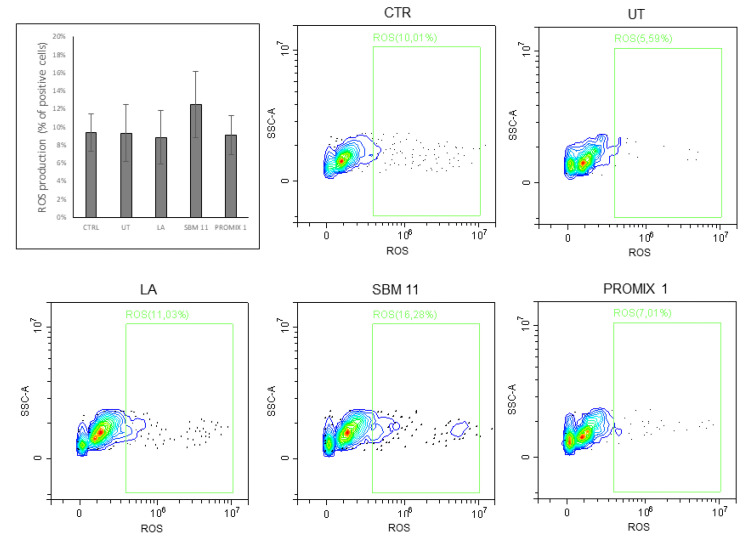
Cytofluorimetric analysis of production of ROS of *Namalwa* cells after incubation with seaweed extract (UT, LA, SBM-11, PROMIX 1) for 24 h compared with untreated control cells (CTR). In the first panel, plot of the percentage of positive cells for ROS production. Data were submitted to one-way analysis of variance (ANOVA), Tukey’s post hoc method was applied to establish significant differences among samples (*p* < 0.05). In the other five panels, representative images of three independent experiments.

In this frame, UT and fermented seaweeds were water extracted and tested in vitro on *Namalwa* cell line for their potential cytotoxicity. This preliminary investigation was directed to assess potential cytotoxic effects induced by the crude aqueous extract of untreated *Gracilaria* and of fermented samples, since microbial treatment could modify the polysaccharide composition. In particular, the viability assay reported in Figure 5 after incubating cells with seaweed water extracts for 24 h revealed cell proliferations statistically similar between UT and treated samples that approached to the CTR. Their biocompatibility was confirmed also by the cell cycle analyses (Figure 6) and the evaluation of ROS production (Figure 7) in *Namalwa* cells incubated with seaweed extracts. As depicted in these figures, both investigated parameters were not statistically affected by the treatment with seaweed aqueous extracts for 24 h, thus evidently confirming cytocompatibility of all seaweed extracts on *Namalwa* cells. These results further support the proposed strategy based on semi-solid fermentation of seaweed as novel route for their stabilization and their subsequent application as novel food.

## 4. Conclusions

In this study, a procedure for stabilizing and processing seaweeds was set up to develop a new treatment of seaweeds for food uses. A biological processing method based on semi-solid fermentation was optimized for the treatment of the seaweed *Gracilaria gracilis* (Gracilariales, Rhodophyta). For the first time, selected LAB and non-conventional coagulase-negative staphylococci were used as starter preparations to obtain new seaweed-based food prototypes for human consumption. The applied starters were able to control the process and to modify the raw material by their specific enzyme profiles.

All treated samples presented a good nutritional value of fatty acids composition. Since LA, has a balanced n-6/n-3 fatty acid ratio; SBM-11 and PROMIX 1 treated samples showed IA and IT recommended values.

Although after fermentation, a significant decrease in bioactive compounds content was observed compared with the untreated raw material, the treated samples still contained relevant amounts of isoprenoids such as vitamin E (α-T) and A (β-carotene and β-cryptoxanthin, provitamin A carotenoids). Therefore, the obtained fermented products are safe and guarantee a good shelf life, suggesting their possible utilization for nutraceutical purposes. This evidence was further supported by preliminary in vitro tests that confirmed the cytocompatibility of *G. gracilis* fermented products.

## 5. Future Perspectives

**Figure 8:**
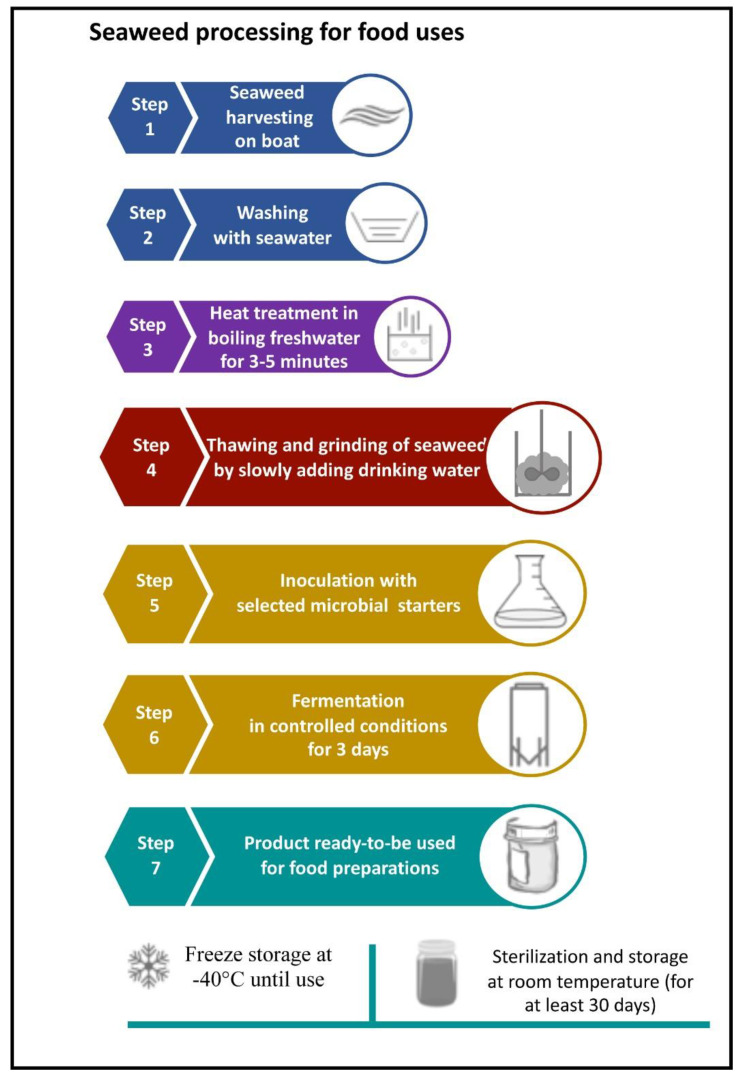
Diagram illustrating the optimized procedure for seaweed processing to obtain new food products.

The optimized procedure for seaweed processing to obtain new food products is illustrated in Figure 8. It was applied to *G. gracilis*, which is already established in several lagoons of the Mediterranean Basin [99,100,101,102,103]. Very recent studies addressed the possible exploitation of the red alga *G. gracilis* [34,104,105].

Fermentation conducted using selected microbial starters could be a promising instrument not only for the stabilization of seaweeds, which are very perishable and prone to spoilage, but also to produce new sensorial characteristics such as aroma, taste and texture as well as to improve the quality (nutrients content), safety and shelf-life of the deriving products. Seaweed-based fermented products were demonstrated also easier to digest and with a favourable biological profile when investigated in vitro. They can also be used as a suitable fermentative substrate for both producing and administering probiotics, and the fermented end products can be exploited as sources of prebiotics and of derived bioactive compounds. All these considerations make fermented seaweeds a valuable and sustainable building block for the formulation of healthy foods, functional foods and also for other preparations.

The method proposed here, initially performed at laboratory scale, could be easily applied to other seaweed species, and after a proper optimization/validation phase including development in collaboration with companies and national/international authorities, it might be easily transferred to the local producers of coastal communities. Additionally, the procedure discussed here may be readily integrated into programs of biomass production in innovative multitrophic systems, as also suggested by Giangrande et al. [106].

This approach can be a driving force for triggering new local bio-based strategies for seaweed exploitation as novel foods, especially in the Mediterranean Basin that suffers of an increasing combination of socioeconomic and environmental drivers of pressure (fishing, tourism, aquaculture, marine litter, climate change, etc.), thus paving the way for the generation of a sustainable, blue-based bio-economy.

## Figures and Tables

**Table 1:** Microbiological analyses of fermented seaweed after 72 h treatment. The different letters in line indicate significant differences between samples (*p* < 0.05). LA: *Lactobacillus acidophilus*; PROMIX 1: Staphylococcus xylosus; SBM-11: a mix of *Lactobacillus sakei*, *Staphylococcus carnosus* and *Staphylococcus xylosus*.

Microorganisms	Medium	Untreated Sample	LA	PROMIX 1	SBM-11
		Mean (CFU/g)	Mean (CFU/g)	Mean (CFU/g)	Mean (CFU/g)
**TBC**	**PCA**	2.2 × 10^8^ ± 6.8 × 10^7^ (a)	9.7 × 10^6^ ± 1.6 × 10^5^ (a)	1.5 v 10^8^ ± 2.6 × 10^7^ (a)	1.1 × 10^7^ ± 1.4 × 10^6^ (a)
	**sNA**	4.5 × 10^8^ ± 6 × 10^7^ (b)	3.9 × 10^6^ ± 2.7 × 10^5^ (b)	9.2 × 10^7^ ± 7.2 × 10^6^ (b)	1.7 × 10^7^ ± 3.4 × 10^6^ (b)
***Bacillus* spp.**	**BCSA**	1.3 × 10^8^ ± 6.5 × 10^7^ (a)	5 × 10^5^ ± 5 × 10^4^ (c)	8 × 10^7^ ± 6.9 × 10^6^ (a)	5.6 × 10^6^ ± 4.2 × 10^5^ (c)
***Bacillus cereus***		0 (c)	0 (d)	0 (c)	0 (d)
**H_2_S-producing bacteria**	**IRON AGAR**	0 (c)	0 (d)	0 (c)	0 (d)
***Clostridium perfringens***	**SPS**	0 (c)	0 (d)	0 (c)	0 (d)
**Enterobacteriaceae**	**VRBGA**	0 (c)	0 (d)	0 (c)	0 (d)
**Coli-Aerogenes Bacteria**	**VRBA**	0 (c)	0 (d)	0 (c)	0 (d)
**Coagulase positive staphylococci**	**Baird Parker Agar**	0 (c)	0(d)	0 (c)	0 (d)
**Pathogenic staphylococci**	**MSA**	0 (c)	0 (d)	0 (c)	0 (d)
***Vibrio* spp.**	**TCBSA**	0 (c)	0 (d)	0 (c)	0 (d)
**Lactic acid bacteria**	**sMRS-Glucose**	1.7 × 10^8^ ± 7.8 × 10^7^ (a)	7.7 × 10^7^ ± 3.6 × 10^6^ (e)	1.8 × 10^8^ ± 1.7 × 10^7^ (a)	1.7 × 10^7^ ± 1.4 × 10^6^ (a)
**Staphylococci**	**sMRS-Sucrose**	1.1 × 10^8^ ± 5.2 × 10^7^ (a)	4.8 × 10^6^ ± 1.1 × 10^5^ (b)	2.3 × 10^8^ ± 2.4 × 10^7^ (d)	1.4 × 10^7^ ± 2.5 × 10^6^ (a, b)
**Yeast/Moulds**	**DRBC**	0 (c)	4 × 10^1^ ± 6.4 × 10^0^ (d)	0 (c)	0 (d)
	**sSDA**	0 (c)	0 (d)	0 (c)	0 (d)

TBC: total bacterial count at 30 °C; CFU: Colony Forming Unit; PCA: plate count agar; sNA: saline Nutrient Agar; BCSA: Bacillus chromoselect agar; TCBSA: Thiosulphate citrate bile sucrose agar; SPS: Sulphite polymyxin sulphadiazine agar; sSDA: saline Sabouraud Dextrose Agar.

**Table 2:** Main parameters chosen for safety analysis of seaweed-based fermented food preparations.

Assays	Limits	Analytical Reference Method	Reference
Aerobic colony count	<10^5^ CFU/g (*for multi-ingredient cooked ready to eat preparations)* <10^6^ CFU/g (*for multi-ingredient not cooked ready to eat preparations*)	[46]	[47,48,49,50]
β-glucuronidase positive *Escherichia coli*	<10 CFU/g	[51]	[47,48]
Enterobacteriaceae	<10^2^ CFU/g	[52]	[53]
Presumptive *Bacillus cereus*	<10^2^ CFU/g	[54]	[53]
*Clostridium perfringens*	<10 CFU/g	[55]	[49,50]
Coliforms	<10 CFU/g <=70 MPN/100 mL	[56]	[57,58]
Coagulase positive *Staphylococci*	<10^2^ CFU/g, 10^2^ < X < 10^3^ CFU/g	[59]	[47,48]
*Vibrio parahaemolyticus*	Absence in 25 g	[60]	[52,61]
*Vibrio cholearae*	Absence in 25 g	[60]	[61]
Moulds and yeasts	<10^2^ CFU/g *(Marinated octopus, seafood cocktail)*	[62,63]	[49]

**Table 3:** Values of pH of fermented seaweed at 0, 24 h, 48 h and 72 h treatment. LA: Lactobacillus acidophilus; SBM-11: a mix of Lactobacillus sakei, Staphylococcus carnosus and Staphylococcus xylosus; PROMIX 1: Staphylococcus xylosus.

pH				
	0	24 h	48 h	72 h
**UT**	8.6	5.82	5.19	6.02
**LA**	8.6	4.9	4.41	4.2
**SMB-11**	8.6	4.96	5.08	4.7
**PROMIX 1**	8.6	5.13	4.87	4.2

UT: Untreated seaweed sample.

**Table 4:** Fatty acids composition (as percentage of total fatty acids, TFA) of untreated control (UT) and in fermented seaweed. LA: *Lactobacillus acidophilus* SBM-11: a mix of *Lactobacillus sakei*, *Staphylococcus carnosus* and *Staphylococcus xylosus*; PROMIX 1: *Staphylococcus xylosus*. Data shown are the mean ± SD (*n* = 3). Asterisk indicates statistically significant differences (*p* < 0.05) between each treatment (LA, PROMIX 1, SBM-11) versus untreated control (UT), as determined by the two sample-*t*-test.

	UT	LA	PROMIX 1	SBM-11
**Lipids % DW**	5.38 ± 0.42 *	5.28 ± 0.36	3.61 ± 1.03 *	5.41 ± 0.41
	**% TFA**			
**SFA**				
Myristic acid (14:0)	3.52 ± 0.24 *	2.46 ± 0.13 *	3.16 ± 0.33	1.23 ± 0.36 *
Palmitic acid (16:0)	21.63 ± 1.91 *	39.10 ± 2.31 *	26.11 ± 1.98 *	20.02 ± 1.85 *
Stearic acid (18:0)	9.20 ± 0.08 *	39.73 ± 3.21 *	18.98 ± 1.81 *	20.06 ± 1.12 *
Docosanoic acid (C22:0)	0.17 ± 0.02 *	0.24 ± 0.01 *	nd	0.23 ± 0.01 *
**Total**	34.52 ± 2.25 *	81.53 ± 5.66 *	48.25 ± 4.12 *	41.54 ± 3.34 *
**MUFA**				
Palmitoleic acid (16:1 n-7)	1.05 ± 0.04 *	0.47 ± 0.02 *	1.01 ± 0.01	0.93 ± 0.02 *
Oleic acid (18:1 n-9)	7.24 ± 0.12 *	2.80 ± 0.11 *	5.13 ± 0.21 *	4.85 ± 0.13 *
Vaccenic acid (18:1 n-7)	1.59 ± 0.01 *	0.65 ± 0.03 *	2.39 ± 0.11 *	1.19 ± 0.01 *
Erucic acid (22:1 n-9)	0.53 ± 0.02	0.48 ± 0.01	0.63 ± 0.01	0.80 ± 0.02
**Total**	10.41 ± 0.19 *	4.40 ± 0.17 *	9.16 ± 0.34 *	7.77 ± 0.18 *
**PUFA**				
4,7,10,13 hexatetranoic acid (16:4 n-3)	0.82 ± 0.03 *	0.72 ± 0.03 *	0.72 ± 0.04 *	0.89 ± 0.03 *
Linoleic acid (18:2 n-6)	4.79 ± 0.21 *	0.38 ± 0.01 *	1.12 ± 0.01 *	4.19 ± 0.51 *
γ-linolenic acid (18:3 n-3)	0.25 ± 0.01 *	0.22 ± 0.01 *	0.40 ± 0.01 *	0.25 ± 0.03
α-linolenic acid (18:3 n-6)	0.19 ± 0.02 *	0.27 ± 0.02 *	nd	0.10 ± 0.01 *
Stearidonic acid (18:4 n-3)	0.49 ± 0.02	0.50 ± 0.03	0.43 ± 0.04	0.48 ± 0.02
Dihomo γ linoleic acid (20:3 n-6)	1.01 ± 0.01 *	0.35 ± 0.01 *	0.86 ± 0.02 *	0.49 ± 0.01 *
Arachidononic acid (20:4 n-6)	46.36 ± 3.21 *	10.68 ± 0.91 *	37.59 ± 1.23 *	43.72 ± 1.54
8,11,14,17 Eicosatrienoic acid (20:4 n-3)	0.08 ± 0.01 *	0.35 ± 0.01 *	0.04 ± 0.01 *	0.07 ± 0.01
Eicosapentaenoic acid (20:5 n-3)	0.73 ± 0.02 *	0.60 ± 0.05 *	1.43 ± 0.01 *	0.50 ± 0.05 *
Adrenic acid (22:4 n-6)	0.35 ± 0.03	nd	nd	Nd
**Total**	55.07 ± 3.57 *	14.07 ± 1.08 *	42.59 ± 1.37 *	50.69 ± 2.21 *
**Nutritional Index**				
P/S	1.59 ± 0.20 *	0.17 ± 0.02 *	0.88 ± 0.10 *	1.22 ± 0.14
n-6/n-3	22.23 ± 1.10 *	4.88 ± 0.66 *	13.10 ± 0.89 *	22.15 ± 2.35
IA	0.54 ± 0.07 *	2.65 ± 0.33 *	0.75 ± 0.09 *	0.43 ± 0.08
IT	0.64 ± 0.08 *	2.05 ± 0.22 *	0.87 ± 0.10 *	0.61 ± 0.09
h/H	2.60 ± 0.37 *	0.44 ± 0.06 *	1.77 ± 0.20 *	2.75 ± 0.40
UI	213.69 ± 13.82 *	59.68 ± 4.48 *	177.45 ± 5.78 *	201.81 ± 9.01

IA: [C12:0 + (4 × C14:0) + C16:0]/UFA; IT: (C14:0 + C16:0/[(0.5 × MUFA) +(0.5 × n-6 PUFA) + (3 × n-3 PUFA) + (n-3/n-6)]; h/H: (MUFA + PUFA)/(C14:0 + C16:0); UI: 1 × (% monoenoics) + 2 × (% dienoics) + 3 × (% trienoics) + 4 × (% tetraenoics) + 5 × (% pentaenoics) where: PUFA = polyunsaturated fatty acids, SFA = saturated fatty acids, UFA = unsaturated fatty acids, MUFA = monounsaturated fatty acids.

**Table 5:** Isoprenoid (tocopherols, carotenoids and chlorophylls) composition of untreated control (UT) and in fermented *Gracilaria gracilis*. LA: *Lactobacillus acidophilus*; SBM-11: a mix of *Lactobacillus sakei*, *Staphylococcus carnosus* and *Staphylococcus xylosus*; PROMIX 1: *Staphylococcus xylosus*. Data shown is the mean ± SD (*n* = 3). Asterisk indicates statistically significant differences (*p* < 0.05) between each treatment (LA, PROMIX 1, SBM-11) versus untreated control of *Gracilaria gracilis* (UT) as determined by the two sample-*t*-test.

	UT	LA	PROMIX 1	SBM-11
	mg/100 g DW			
**Tocopherols**				
α-T	3.90 ± 0.23 *	nd	0.38 ± 0.10 *	1.56 ± 0.31 *
**Carotenoids**				
Violaxanthin	0.71 ± 0.03	nd	nd	Nd
Fucoxanthin	8.60 ± 0.06 *	0.82 ± 0.07 *	1.09 ± 0.19 *	0.73 ± 0.14 *
Lutein	0.39 ± 0.04	0.36 ± 0.04	0.37 ± 0.12	0.37 ± 0.11
Zeaxanthin	16.45 ± 0.33 *	5.47 ± 0.17 *	7.98 ± 0.47 *	9.29 ± 0.94 *
α-cryptoxanthin	13.26 ± 1.03 *	2.71 ± 0.03 *	4.64 ± 0.38 *	4.24 ± 0.98 *
β-cryptoxanthin	14.19 ± 0.44 *	2.52 ± 0.21 *	5.70 ± 0.38 *	6.40 ± 0.40*
β-carotene	9.26 ± 0.99 *	1.50 ± 0.20 *	4.06 ± 0.03 *	5.26 ± 0.15 *
9 cis β-carotene	1.73 ± 0.42 *	0.34 ± 0.01 *	0.49 ± 0.01 *	0.39 ± 0.06 *
***Total***	64.59 ± 3.33 *	13.72 ± 0.73 *	24.33 ± 0.42 *	26.68 ± 2.78 *
Chlorophylls a + b	60.93 ± 1.61 *	21.49 ± 0.60 *	37.60 ± 2.78 *	26.75 ± 3.51 *

## Data Availability

Data are contained within the article.
